# Development of an impedimetric aptasensor for Shiga-like toxin 1 using a DNA aptamer and an interdigitated microelectrode

**DOI:** 10.1039/d5ra09084a

**Published:** 2026-01-21

**Authors:** Malaya Mili, Somasekhar R. Chinnadayyala, Sungbo Cho, Pranab Goswami

**Affiliations:** a Department of Biosciences and Bioengineering, Indian Institute of Technology Guwahati Guwahati 781039 Assam India pgoswami@iitg.ac.in; b Department of Electronic Engineering, Department of Semiconductor Engineering, Gachon University Seongnam-si Gyeonggi-do 13120 Republic of Korea; c Gachon Advanced Institute for Health Science & Technology, Gachon University Incheon 21999 Republic of Korea

## Abstract

The life-threatening diarrheal disease caused by Shiga toxin-producing *Escherichia coli* (STEC) necessitates rapid, sensitive, portable, and low-cost diagnostic tools to strengthen on-field food safety monitoring, improve disease prognosis, and guide timely therapeutic interventions. In this study, an impedimetric aptasensor was developed against Shiga like toxin-1 produced by STEC using a 71-mer ssDNA aptamer (sT3), enriched from an initial library (∼10^14^ sequences) through the Systematic Evolution of Ligands by Exponential Enrichment (SELEX) technique, and an interdigitated chain-type microelectrode fabricated by depositing Ti and Au layers on a glass substrate *via* electron-beam deposition, photolithography, and wet-chemical etching. The aptamer exhibited a strong binding affinity with a dissociation constant (*K*_d_) of 273 ± 0.03 nM, as determined by isothermal titration calorimetry. The resulting aptasensor achieved a detection limit of 2.88 pM, a linear dynamic range of 10–450 pM, and a sensitivity of 107.02 Ω pM^−1^, underscoring its strong analytical performance. It retained 97.6% of its initial response after 25 days with five intermittent operations and showed high reproducibility, with only 3.9% standard deviation across five independently fabricated sensors. These stable performances were attributed to the strong aptamer–surface coupling enacted by covalent immobilization through a self-assembled monolayer strategy. The aptasensor further demonstrated high selectivity and practical applicability by reliably detecting the toxin protein in spiked milk samples. Overall, the developed aptasensor offer a great prospect for its practical application as a diagnostic device for rapid and reliable detection of Shiga toxin-induced gastroenteritis at point-of-care settings.

## Abstract 2

Life-threatening diarrheal disease caused by Shiga toxin-producing *E. coli* demands rapid, sensitive, portable, and low-cost diagnostics to enhance on-site food safety monitoring, improve prognosis, and enable timely therapeutic intervention.

## Graphical abstract

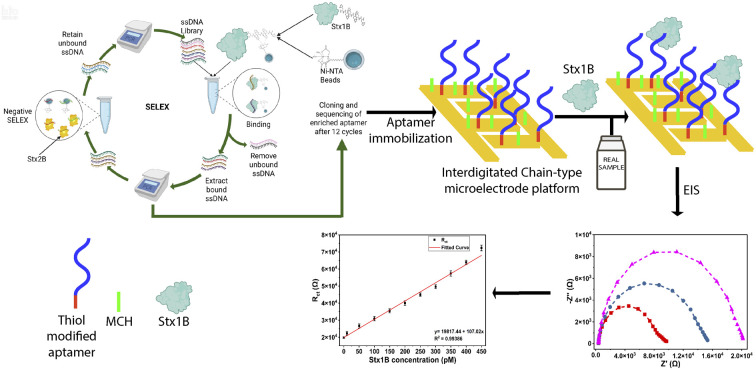

## 1. Introduction

Shiga toxin-producing *E. coli* (STEC) has caused numerous outbreaks worldwide, resulting in significant mortality and morbidity.^1^ Infection with STEC leads to gastroenteritis, causing bloody diarrhea, hemorrhagic colitis, and hemolytic uremic syndrome. Children under 5 years of age and elderly people over 65 years of age are more susceptible to infection by STEC. The Shiga toxins produced by STEC are considered the primary virulence factor. They produce two types of toxin, Shiga-like toxin 1 (Stx1) and Shiga-like toxin 2 (Stx2). Both Stx1 and Stx2 are responsible for the pathogenicity of STEC, but they differ in their amino acid sequences with sequence similarity of ∼56%,^2^ receptor affinities, and immunological characteristics.^3^ The Shiga toxins have an AB_5_ structure, with one A subunit bearing the enzymatic active site and a pentameric B subunit, used for binding to the glycolipid globotriaosylceramide (Gb_3_) on the target cells. The catalytic A subunit exhibits *N*-glycosidase activity, which removes an adenine residue at position 4324 of the 28S rRNA, thereby halting the protein synthesis mechanism at the target cell.^4^ The molecular weight of the A subunit of the Shiga toxins is ∼32 kDa, and each B subunit is ∼7.7 kDa.

Multiple routes of transmission for STEC are known, such as contaminated food and water, contact with animals, and person-to-person interactions.^5^ The source of most STEC outbreaks has been identified as cattle meat and its dairy products, tap water (including well water), fruits and vegetables, and their products.^6^ Currently, treatment for STEC infection involves palliative care, which includes prompt fluid replacement, management of electrolyte imbalances, and control of elevated blood pressure.^7^ Treatment with antibiotics is generally not recommended for STEC-infected patients, as studies have reported conflicting outcomes. While some investigations indicate an increased risk of hemolytic uremic syndrome,^8^ others have found no significant association, or even a potential benefit, depending on the class of antibiotic and treatment regimen employed.^9,10^ The sequence and structure of Stx1 are nearly identical to those of the Shiga toxin (Stx) produced by *Shigella dysenteriae* type 1, making its detection crucial for accurate diagnosis, strain differentiation, and food safety surveillance.^11^ Therefore, there is a critical need for a rapid, low-cost, and accurate point-of-care diagnostic method to strengthen food safety monitoring, improve disease prognosis, and guide appropriate therapeutic interventions.

The present study focuses on the detection of Stx1, for which several analytical methods have been reported, including conventional culture-based assays,^12^ PCR-based techniques,^13^ ELISA-based approaches,^14^ and antibody-based biosensors.^15^ However, these methods involve complex procedures, require long assay times and skilled personnel, and often depend on expensive instruments. Moreover, antibody-based detection systems suffer from inherent limitations such as high production costs and poor stability, as antibodies readily lose their activity with changes in temperature, pH, or storage conditions, making them less suitable for field-based or point-of-care applications.

Aptamers offer a promising alternative to antibodies owing to their superior thermal and chemical stability, lower production costs, and ease of large-scale synthesis.^16,17^ These single-stranded oligonucleotides are typically developed through the Systematic Evolution of Ligands by Exponential Enrichment (SELEX) process. Their facile functionalization and high design flexibility allow the aptamers to be interfaced with diverse transduction platforms to construct biosensors capable of converting target recognition into quantifiable electrical,^18^ optical,^19^ or colorimetric^20^ signals.^16^ Several electrochemical sensors have been developed for detection of toxins such as aflatoxin,^21^ fumonisin B1,^22^ ochratoxin A^23^ demonstrating sensitive electrochemical-based detection of toxins. While many reported sensors rely on complex modification or multi-step amplification process to achieve high sensitivity, there remains a need for label free, low cost, simple, Point of care (POC) deployable sensor for Shiga like toxin detection.

Interdigitated microelectrodes are known for their portability and low-cost fabrication, making them highly suitable for POC applications. These microdevices require minimal sample volume, provide a high signal-to-noise ratio, and exhibit low response times.^24^ In a typical rectangular-shaped electrode interdigitated array, a strong electric field appears at the edge of the fingers, making the sensing area non-homogeneous. To avoid the edge effect, designing the electrode for a homogeneous electric field distribution in the sensing area can lead to better and more reliable electric signals.^25^

In this work, an interdigitated chain-shaped microelectrode (ICE) with no sharp edges was fabricated to achieve a homogeneous electric field distribution within the sensing area. A high-affinity aptamer targeting the Stx1-B subunit was developed using the SELEX technique and subsequently immobilized on the ICE to construct an electrochemical impedance spectroscopy (EIS)-based Stx1 biosensor. When coupled with EIS as the detection principle, the system monitors changes in charge transfer resistance, double-layer capacitance, and dielectric properties upon target binding at the electrode surface, enabling highly sensitive detection of the biomarker.^26^ A detailed account of the findings is presented in this paper.

## 2. Experimental

### 2.1 Materials

The Stx1B and Stx2B gene sequences were synthesized by Genscript (USA). Biotinylated reverse primer, forward primers, and ssDNA library with the sequence CACCTAATACGACTCACTATAG-N30-CTGGCTCGAACAAGCTTGC, for SELEX were procured from IDT (USA). 5′-SH-(CH_2_)_6_-CACCTAATACGACTCACTATAGACTAAAGGGGCTTGATACCATAACTATGTCCTGGCTCGAACAAGCTTGC-3′ thiol-modified sequence was procured from Eurofins (Sweden). Streptavidin magnetic particles were purchased from Roche (Germany). Ni-NTA agarose beads were procured from Qiagen. Emerald Amp® GT PCR Master mix was procured from DSS Takara Bio (India). All the reagents used were of analytical reagent grade, and the buffers were prepared using deionized water (18.2 MΩ cm).

### 2.2 Cloning, expression, purification, and characterization of Stx1B and Stx2B

The Stx1B and Stx2B nucleotide sequences were curated from the National Centre for Biotechnology Information (NCBI) (accession number AE005174) and commercially synthesized in the pGEM-T vector after codon optimization. The sequences were then cloned into pET-28(a) vector using Ncol and Xhol as the restriction sites and transformed into *E. coli* DH5α cells for storage. The recombinant pET-28(a)-Stx1B plasmid was isolated and transformed into *E. coli* BL21 (DE3) pLysS cells and the pET-28(a)-Stx2B plasmid was isolated and transformed into Rosetta 2 (DE3) cells for protein expression. Further, the recombinant Stx1B protein with a His tag at the C-terminal was expressed using 0.2 mM IPTG induction at 16 °C, and the recombinant Stx2B was expressed with a His tag at the C-terminal with 0.2 mM IPTG induction at 37 °C in 1 × PBS buffer. The expressed proteins were purified using a Ni-NTA affinity column (Cytiva, USA) and visualized in tricine SDS-PAGE. A western blot was then performed to confirm the presence of the protein. An anti-Stx1B monoclonal antibody (Sigma, Germany) was used to detect the Stx1B protein, and an anti-His antibody (RealGene, India) was used to detect the Stx2B protein. Additionally, the molecular weights of the purified proteins were determined using MALDI-MS (Bruker, USA), and their secondary structure compositions were estimated through circular dichroism (CD) (Jasco, Japan) studies.

### 2.3. SELEX studies

**Fig. 1:**
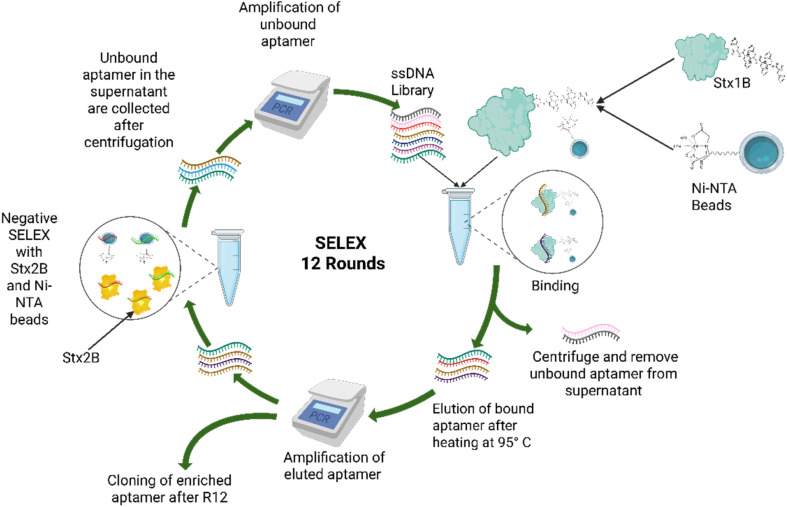
Schematic representation of the SELEX process used for aptamer selection (created in https://BioRender.com).

The SELEX process was carried out by immobilizing the His-tagged Stx1B and the Stx2B on Ni-NTA agarose beads (Fig. 1). For immobilization of the His-tagged protein with Ni-NTA agarose beads, the beads were washed three times with the Ni-NTA binding buffer (200 mM NaH_2_PO_4_, 500 mM NaCl, and 5 mM imidazole, pH 7.4). Subsequently, 100 µL of 10 µM protein was incubated with 100 µL of Ni-NTA agarose beads in the same binding buffer for 30 minutes at room temperature with a gentle mixing on a rocker platform at 30 rpm. After incubation, the protein-bound beads were washed three times with SELEX binding buffer (50 mM sodium phosphate, 50 mM NaCl, 5 mM KCl, and 2.5 mM MgCl_2_) to remove any unbound protein, and then the beads were resuspended in the same buffer.

Before starting the SELEX cycle, 10 µM of the ssDNA library (∼10^14^ sequences) was added to 50 µL of SELEX binding buffer and heated at 95 °C for 10 minutes, and immediately cooled on ice for 10 minutes. The initial two cycles of the SELEX process were negative SELEX. In the first cycle, the ssDNA library was incubated with only the Ni-NTA agarose beads in a microcentrifuge tube for one hour at room temperature with a gentle mixing on a rocker platform at 30 rpm, to discard the sequences that have a binding affinity for the beads. To separate the unbound aptamers from the beads binding aptamers, the tube was centrifuged at 1500 × g for two minutes. The unbound aptamers remained in the supernatant, and the aptamers binding to the Ni-NTA beads were pelleted down along with the beads. The aptamers in the supernatant were collected and amplified by PCR to use as the ssDNA library in the next cycle. In the second cycle, the Ni-NTA agarose bead-bound Stx2B protein was used as a negative target, as it has a nearly similar structure to Stx1B, and was incubated with the ssDNA library for one hour at room temperature with a gentle mixing on a rocker platform at 30 rpm. Following the incubation, the tube was centrifuged, and the supernatant was collected and used in the next cycle as the library. From the third round, the positive SELEX was performed, where the curated library was allowed to interact with the Ni-NTA bead-bound Stx1B for one hour at room temperature with gentle agitation on a rocker platform at 30 rpm. Following the incubation, the mixture was centrifuged, and the pellet containing the aptamers bound to the Stx1B was collected. To elute the Stx1B bound aptamers, the pellet was resuspended in SELEX binding buffer and heated at 95 °C for 10 minutes and centrifuged at 1500 × *g* for two minutes. Following the centrifugation, the supernatant was collected and amplified with PCR to be used as the ssDNA library in the next cycle. In cycles 1 and 7, Ni-NTA beads were allowed to interact with the ssDNA library, and in cycles 2 and 8, Stx2B was allowed to interact with the ssDNA library. A total of 12 cycles of SELEX were performed, out of which four cycles were negative SELEX and eight were positive SELEX cycles. The time duration for protein aptamer interaction during positive SELEX was incrementally reduced from 60 minutes for R3 and R4 to 45 minutes for R5 and R6, and then to 30 minutes for R9 and R10, and to 15 minutes for R11 and R12. For negative SELEX, the incubation time was increased from 60 minutes for R1 and R2 to 90 minutes for R7 and R8 (Table S1). After completing 12 cycles, the PCR amplicons were used for cloning.

### 2.4 Cloning of aptamer sequences

After completing the 12 SELEX cycles, the enriched ssDNA pool was subjected to PCR amplification. The amplified products were purified using a PCR purification kit (Gene2protein, India) and subsequently cloned into the pGEM-T vector, which was then transformed into *E. coli* DH5α cells. The transformed cells were spread on LB agar plates containing kanamycin as an antibiotic, along with X-gal and isopropyl β-d-1-thiogalactopyranoside (IPTG) for blue white screening of transformed colonies. The white colonies from the agar plates were picked and grown in LB media, and their plasmids were isolated using a plasmid isolation kit (Gene2Protein, India). The isolated plasmids were sequenced *via* Sanger sequencing.

### 2.5 Prediction of aptamer structure and docking

The secondary and tertiary structures of the selected sequences were computationally predicted and then docked with Stx1 to identify the interacting residues. The secondary structures were predicted using the Mfold web server (https://www.unafold.org/mfold/applications/dna-folding-form.php),^27^ at 25 °C and 150 mM Na^+^ concentration. The server predicts the secondary structure along with the free energy (Δ*G*) of an ssDNA from its sequence. The dot-bracket format of the secondary structure was computed using the RNAComposer website, which was used to predict the tertiary structure.^28^ The tertiary structures were predicted using the 3dRNA/DNA web server (http://biophy.hust.edu.cn/new/3dRNA/create) using the default procedure.^29^ Furthermore, the tertiary structure of the aptamer sequence was docked with the crystal structure of Stx1 using the HDOCK server at default conditions.^30^ To study the molecular interaction between the protein and the aptamer, the docked structures were analyzed using the Protein–Ligand Interaction Profile (PLIP) web server.^31^ It identifies and characterizes the various non-covalent interactions between the protein and the aptamer.

### 2.6 Isothermal titration calorimetry (ITC)

The binding interaction of the aptamer and the protein was analysed using ITC (iTC 200 microcalorimeter from GE Healthcare). Both the protein and the aptamer were prepared in binding buffer (50 mM sodium phosphate, 50 mM NaCl, 5 mM KCl, and 2.5 mM MgCl_2_). The protein, at a concentration of 20 µM, was loaded into the syringe, and the aptamer, at a concentration of 1.5 µM, was loaded into the cell. An initial volume of 0.4 µL of protein solution was injected, followed by 39 injections of 1 µL of protein solution at 120 second intervals, and the solutions were mixed in the reaction cell at 600 rpm. Further, the data were analysed using the Origin software.

### 2.7 Fabrication of ICE

**Fig. 2:**
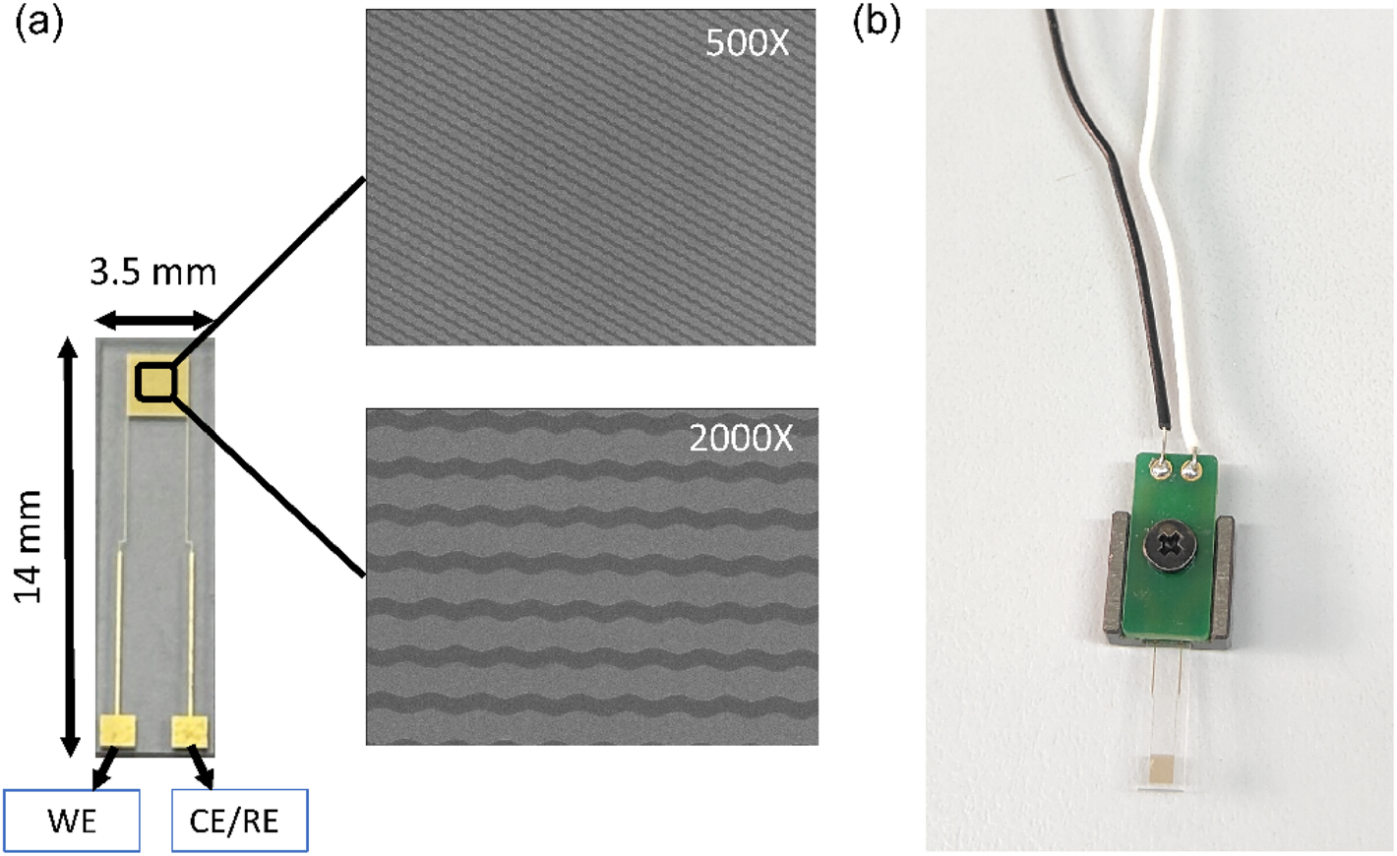
(a) FESEM image of ICE with 500× and 2000× magnification. (b) Adapter for connecting the ICE with the potentiostat.

An interdigitated chain-type microelectrode (ICE) was fabricated on a glass slide with dimensions of 14 mm × 3.5 mm. A conductive layer of Ti and Au was deposited with a thickness of 25 nm and 50 nm, respectively, *via* an electron beam depositor. Subsequently, photolithography and a chemical wet etching process were employed to create interdigitated chain-type electrode fingers with a 5 µm spacing and width, as well as contact pads for the working electrode and the reference electrode. The FESEM image of the ICE was captured with the sensing arrays, showing the interdigitated electrode fingers and contact pads (Fig. 2). The ICE was connected to the potentiostat (Zahner, Germany) using a home-made adapter.

### 2.8 Immobilization of aptamers on interdigitated chain-type microelectrode (ICE)

The thiol-modified aptamer was immobilized on the Au-based ICE *via* covalent bonding. Prior to immobilization of the aptamers, tris(2-carboxyethyl) phosphine chloride (TCEP) was used to reduce the protective disulfide group on the thiol-modified aptamers to expose the free thiol group (-SH). The thiol-modified aptamer was treated with TCEP at a molar ratio of 1 : 100 (aptamer: TCEP) and incubated for 1.5 hours at room temperature to ensure the complete reduction of the disulfide linkage. Further, the reduced aptamers were purified using the ethanol precipitation method. The ICE was cleaned using 0.5 M NaBH_4_, washed with DI water, and dried using a stream of Argon gas. The sensing area on the ICE containing the Au electrode fingers was dipped in a solution of reduced thiol-modified aptamers and kept at 4 °C overnight. The aptamer-bound electrode was washed with binding buffer to remove any unbound or loosely bound aptamers, and then backfilled with a 1-mercapto-6-hexanol solution (MCH). Finally, the electrodes were rinsed with DI water and kept at 4 °C until use.

### 2.9 ICE surface characterization

To confirm the immobilization of the aptamers on the electrode surface, the electrode surface was examined using Atomic Force Microscopy (AFM), which is used to study the changes in surface roughness and morphology of both bare and modified electrodes. It was performed with the Cypher S atomic force microscope (Oxford Instruments, USA). The images were recorded in non-contact mode over a 1 × 1 µm^2^ area.

### 2.10 Electrochemical impedance spectroscopy study

EIS was used to probe the change in the electrode surface after successive modifications and also as the sensing mechanism for the developed biosensor. EIS measurements were performed using a potentiostat (Zennium, Zahner, Germany). The charge transfer resistance (*R*_ct_) value was used to measure the change in charge transfer at the electrode surface. The EIS was carried out in a pseudo-reference setup, with one arm of the ICE serving as the working electrode, the other arm as the counter and the pseudo-reference electrode. A solution containing 5 mM K_3_Fe(CN)_6_/K_4_Fe(CN)_6_ in 1× PBS, pH 7.4, was used as the redox probe. A small AC potential of 5 mV with 0 DC bias was applied and scanned over a frequency range of 1 Hz to 100 kHz with an increment of 5 frequencies per decade. The measurements were performed by dipping the sensing area of the electrode in a well of a 96-well plate containing 350 µL of redox probe solution.

### 2.11 Real sample analysis

The analytical performance of the developed biosensor was further validated using real samples. Commercially purchased milk (Amul, India) was spiked with different concentrations of Stx1B. Prior to measurement, the Stx1B spiked milk sample was centrifuged at 12 000× *g* for 15 minutes at 4 °C, and the upper fat layer was discarded. The measured response of the spiked samples was compared with the calibration curve prepared under buffer conditions. The recovery percentage and the relative standard deviation (RSD) were calculated to determine the accuracy and reproducibility of the sensor.

### 2.12 Statistical analysis of data

All the experiments were performed with a minimum of three independent replicates, and the mean of the measured data was presented with standard deviation. Origin Pro software was used to analyze and generate graphical data. The limit of detection (LOD) of the developed sensor was calculated using the formula, LOD = 3× standard deviation of blank/sensitivity (slope of the calibration curve).^32^

## 3. Results and discussions

### 3.1 Development of aptamer specific to Stx-1B

The nucleotide sequences of the biomarker proteins Stx1B, along with Stx2B (used as a negative control for the SELEX studies), were cloned, expressed, purified, and characterized following the methods described in Section 2.2. The purity of the proteins, as observed in the SDS-PAGE (Fig. S1a), was further validated by western blotting (Fig. S1b). A single band at ∼10 kDa was observed for both proteins in the blot, confirming the successful expression of the proteins. MALDI-TOF analysis confirmed the molecular weights of the purified proteins, revealing that Stx1B and Stx2B are 9 kDa and 8.8 kDa, respectively (Fig. S1d). Additionally, the secondary structure compositions of the proteins were analyzed by CD studies (Fig. S1c). The Stx1B protein contains 11.2% α helix, 43% β sheets, and 45.8% random coils, while Stx2B has a composition of 13% α helix, 39% β sheets, and 48% random coils. These purified proteins were then used in SELEX studies to isolate a specific aptamer for Stx1B. A total of 12 rounds of SELEX cycles were performed, out of which 4 rounds were negative SELEX using Ni-NTA beads and Stx2B protein to eliminate all the aptamer sequences that have a binding affinity for these entities. Following the negative SELEX round, the remaining sequences in the library were subjected to eight rounds of positive SELEX analysis to identify aptamer candidates with strong binding affinity for the target molecule, *i.e.*, Stx1B. PCR was performed at the end of each cycle, and the intensity of the amplified bands was monitored on the gels (Fig. S2). The presence of amplified bands at the expected size after each round of SELEX confirmed the successful recovery and amplification of the ssDNA library. The high intensity of the amplified band after the R1 cycle can be attributed to the high concentration of ssDNA in the starting library leading to recovery of weakly bound and non-specific binders. The intensity of the ssDNA library amplified bands after each cycle was less in the earlier cycles, but gradually towards the later cycles of SELEX process, the intensity of the amplified bands were higher, indicating enrichment of the ssDNA. The intensity of the bands remained constant towards the last rounds of the cycles. With no further enrichment observed, the SELEX process was stopped after 12 rounds. The enriched aptamer candidates of the final positive cycle were then cloned into pGEM-T vector and transformed into *E. coli* DH5α cells and plated on agar plates. A total of 20 random colonies were selected, and plasmids were isolated from each. Among these, 17 colonies were confirmed to contain the desired insert by PCR screening. Plasmids from the PCR-positive colonies were subsequently sequenced to characterize the insert sequences. The obtained sequences were subjected to multiple sequence alignment to identify duplicate or recurring sequences. Among the pool, sequences sT3 and sT12 showed the highest representation, appearing four and two times, respectively. Based on their high relative abundance, these two aptamer candidates were considered for further study.

### 3.2 Aptamer protein interactions

**Fig. 3:**
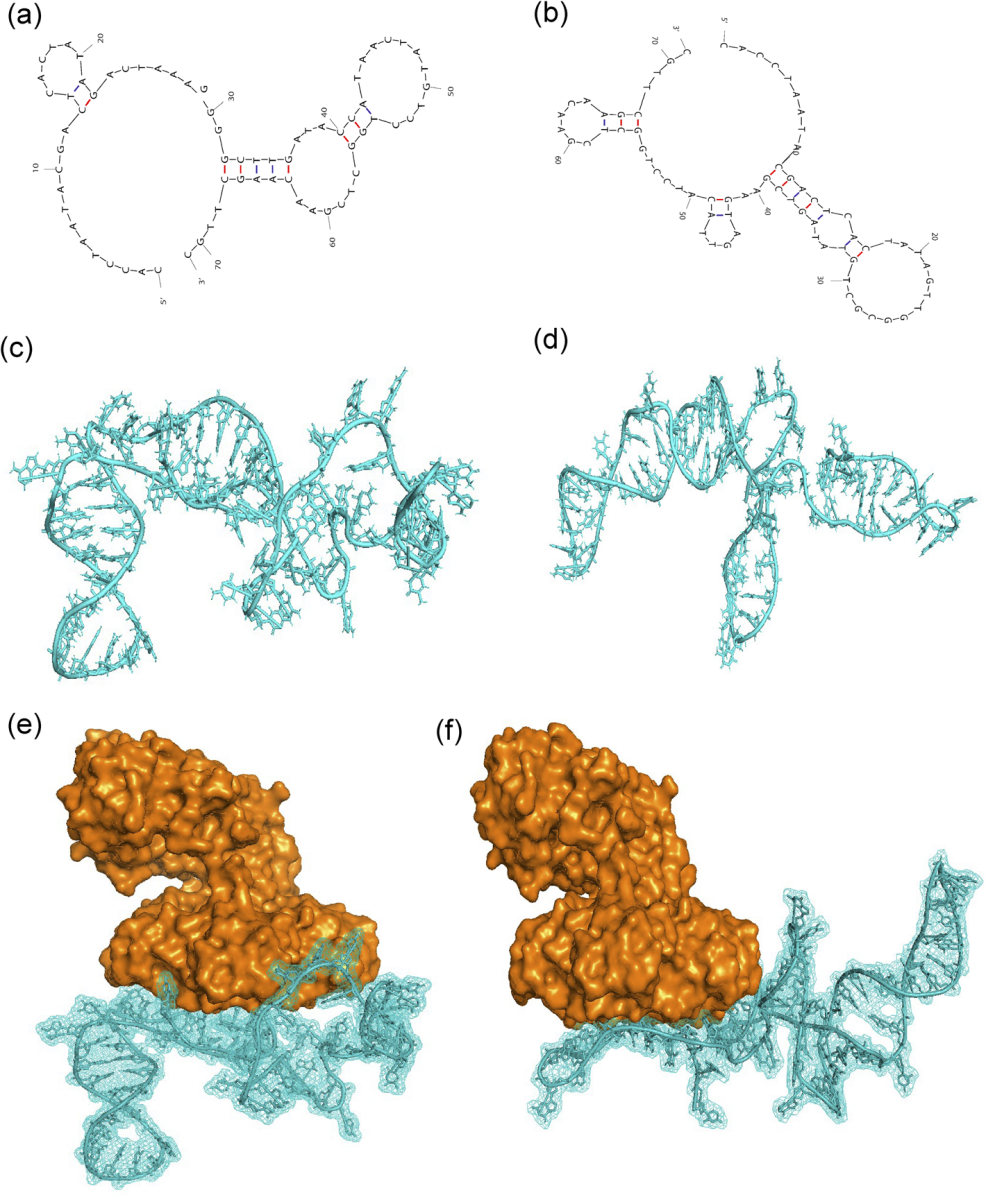
Secondary structures of (a) sT3, (b) sT12, tertiary structures of (c) sT3, (d) sT12, docked structure of (e) Stx1-sT3, and (f) Stx1-sT12.

The secondary structures of the aptamer candidates, sT3 and sT12 were predicted, yielding Δ*G* values of −3.98 kcal mol^−1^ and −7.15 kcal mol^−1^, respectively. Subsequently, their three-dimensional structures were modelled and docked with the Stx1 protein (PDB ID- 1R4Q) using the HDOCK server. The docking results indicated that both sT3 and sT12 interact specifically with the B subunit of Stx1 (Fig. 3). The docked complexes were further analyzed using the PLIP web server, and the interaction details are summarized in Table 1.

**Table 1:** Interaction profile in the aptamer–protein complex, expressed as the ratio between protein residues and aptamer residues

Aptamer–protein complex	H bonds	Hydrophobic bond	π-Cation	π-Stackings	Salt bridges
sT3-Stx1	THR1 : G11	TRP34 : T69	LYS53 : A9	TRP34 : A62	ASP18 : G60
	ASP3 : A9	PHE30 : C71		PHE30 : C71	HIS58 : T8
	ASP3 : C10				
	ASP16 : T58				
	ASP16 : T59				
	ASP16 : G60				
	ASN32 : A61				
	TRP34 : A62				
	TRP34 : A64				
	ASN35 : A65				
	ASN35 : C63				
	LYS53 : A9				
	ASN59 : T8				
	THR290 : A64				
sT12-Stx1	ASN15 : A2	ASP17 : C3	TRP34 : G66	TRP34 : A64	ASP18 : G55
	ASP17 : C3			TRP34 : A65	ARG33 : T58
	ASP18 : T58			HIS58 : A50	
	ASN32 : T54				
	ASN32 : C57				
	ARG33 : T54				
	ARG33 : T5				
	TRP34 : T69				
	THR54 : T5				
	ASN55 : T5				
	GLY62 : T5				
	SER64 : T54				

ITC was performed to determine the dissociation constants (*K*_d_) of the aptamer candidates. The ITC analysis revealed an exothermic interaction between the aptamer and the protein, the data were fitted using a one site binding model, yielding a stoichiometry of *N* = 0.896 ± 0.019 sites and a *K*_d_ values of 273 ± 0.03 nM for sT3 and for sT12 the fitting yielded a stoichiometry of *N* = 0.878 ± 0.175 sites and a *K*_*d*_ values of 6.80 ± 0.56 µM (Fig. S3). These results indicate that sT3 has a significantly higher binding affinity toward the Stx1B subunit. Consequently, sT3 with sequence 5′-CACCTAATACGACTCACTATAGACTAAAGGGGCTTGATACCATAACTATGTCCTGGCTCGAACAAGCTTGC-3’ (Table S2) was selected as the recognition element for the development of the Stx1 electrochemical biosensor using the interdigitated chain-type electrode.

### 3.3 Development of aptamer-immobilized interdigitated electrode

**Fig. 4:**
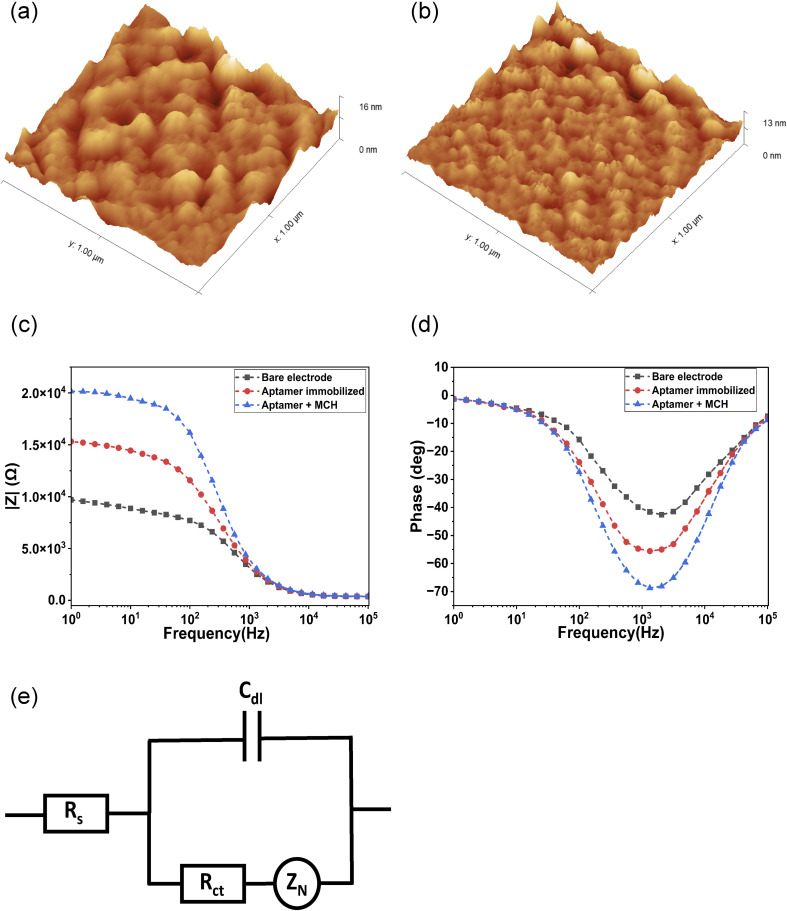
AFM image of the (a) bare electrode, (b) aptamer immobilized electrode. Bode plot of the (c) impedance magnitude (|*Z*|), and (d) phase at different modification stages of the ICE surface. (e) Randle's equivalent circuit for the EIS plots.

The sT3 aptamer was thiol-modified and immobilized onto the surface of a chain-shaped interdigitated gold microelectrode following a self-assembled monolayer (SAM)-based approach. Following the immobilization, the surface morphology of the electrode was characterized by AFM. The bare electrode exhibited a mean roughness of 1.004 nm and a maximum roughness of 15.54 nm. After aptamer immobilization, these values decreased to 0.774 nm and 12.67 nm, respectively. The reduction in surface roughness confirms the formation of a uniform and compact aptamer-based SAM on the electrode surface (Fig. 4a and b).

EIS was employed to analyze the changes on the electrode surface after immobilizing the thiol-modified aptamer and subsequent backfilling with EIS. A redox solution containing 5 mM K_3_Fe(CN)_6_/K_4_Fe(CN)_6_ in 1× PBS, pH 7.4, was used to measure the impedance after each modification. The EIS spectra were plotted as a Bode plot (Fig. 4c and d), and Zahner analysis software was used to fit the spectra with an equivalent circuit model. A Randle's circuit model containing a solution resistance (*R*_s_) connected in series with a combination of Constant Phase Element (CPE) connected in parallel to a combination of charge transfer resistance (*R*_ct_) connected in series with a finite diffusion element represented by a Nernst diffusion element (*Z*_N_), was used to fit the EIS spectra (Fig. 4e).

From Fig. 4c, the distinct regions corresponding to different electrochemical processes can be observed. The higher frequency region above 10 kHz corresponds to the solution resistance (*R*_s_), the higher mid frequency region corresponds to the double layer capacitance (*C*_dl_), the lower mid frequency region around 500 Hz corresponds to the charge transfer resistance (*R*_ct_), and the lowest frequency region corresponds to the Nernst diffusion element (*Z*_N_). The parameters of each electrochemical process, determined by fitting the measured impedance spectra after every modification of the electrode, are given in Table 2. The observed increase in the impedance magnitude |*Z*| after immobilization of the aptamer on the electrode surface indicates the successful formation of a thiol-modified aptamer-functionalized SAM on the electrode surface. The immobilization of the thiol modified aptamer on the gold electrode surface occurs through the formation of a strong Au–S covalent bond, where the sulfur from the thiol group bonds coordinatively with gold atoms, developing a stable and well-defined aptamer layer.^33^ The aptamer layer creates steric hindrance, while the negatively charged phosphate backbone further repels the negatively charged [Fe(CN)_6_]^3−/4−^ ions from reaching the electrode surface, thereby increasing the interfacial resistance. Subsequent backfilling of the aptamer-modified electrode with MCH further blocks the unoccupied spaces on the electrode surface, prevents non-specific adsorption of part of the aptamer due to the repulsion between the negatively charged DNA backbone and the alcohol terminus of the MCH, ensuring the upright orientation of the immobilized aptamers, and regulating the packing of the aptamer density.^33^ The MCH layer further impedes the [Fe(CN)_6_]^3−/4−^ ions from reaching the electrode surface, leading to an increase in the overall impedance magnitude |*Z*|. The *R*_ct_, which represents the resistance to the transfer of electrons between the redox molecules and the electrode surface, also increased with every modification of the electrode surface. The progressive increase in the *R*_ct_ value indicates the successful immobilization of the aptamer and backfilling with MCH. Consequently, the *R*_ct_ value was used to characterize the sensor response in relation to target binding.

**Table 2:** Fitted EIS parameters for bare, aptamer-immobilized, and MCH-blocked electrode

Electrodes	*R*_s_ (Ω)	CPE		*R*_ct_ (kΩ)	*Z*_N_	
		*Y*_0_ (nS s^a^)	*α*		DW (KDW)	*k* (s^−1^)
Bare	362	89.1	928	7.78	6.53	26.6
Aptamer	361	86.7	934	12.3	8.16	24.5
MCH	382	71.0	936	18.8	4.61	14.9

a *Y*_0_ – CPE admittance, α – phase shift factor, DW – warburg constant, *k* – electron transfer rate constant.

### 3.4 Application of the aptamer-immobilized electrode for sensing Stx1-B

**Fig. 5:**
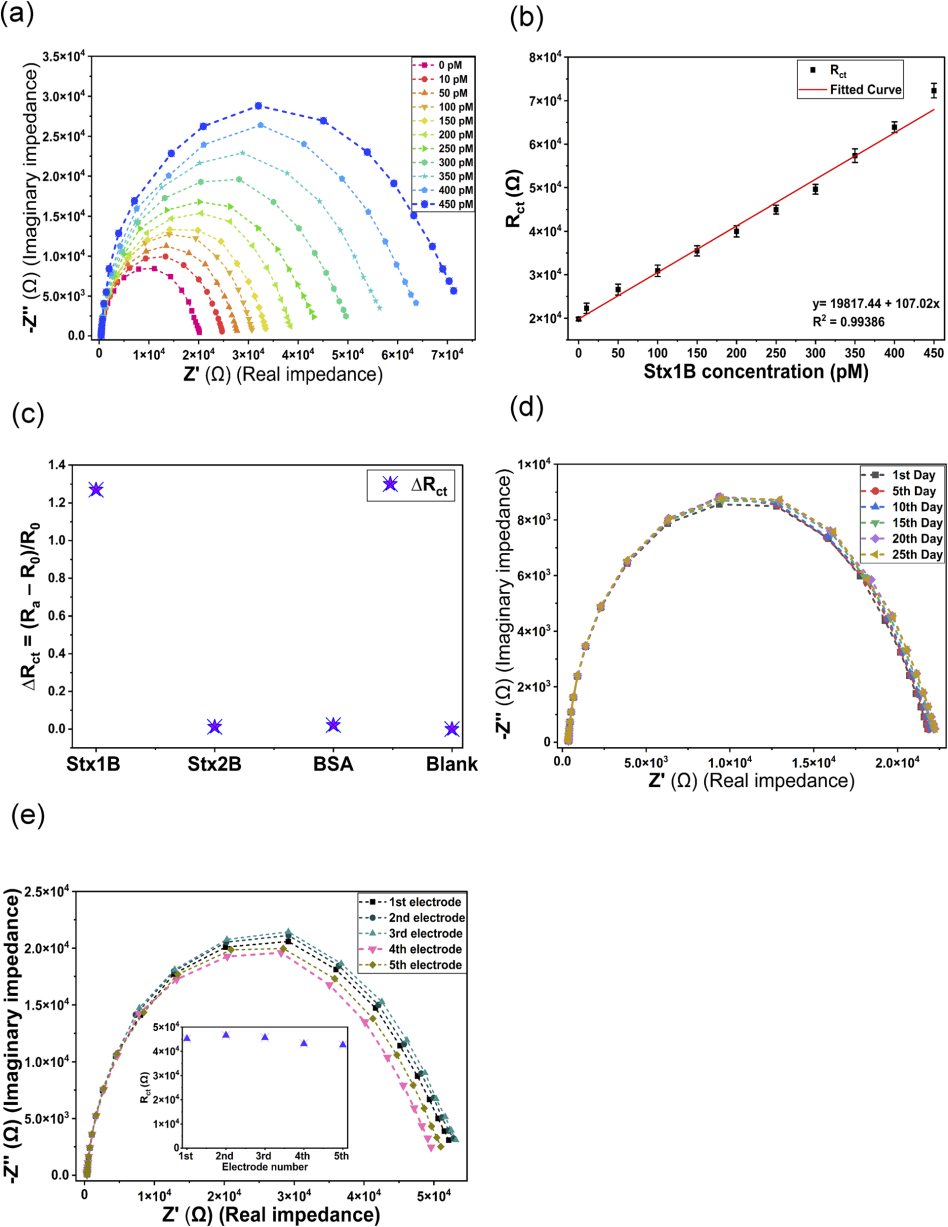
: (a) EIS spectra of the aptasensor with increasing concentration of Stx1B, (b) linear calibration plot using the *R*_ct_*versus* concentration of Stx1B, (c) selectivity analysis of the developed aptasensor. (d) Stability analysis over 25 days, (e) reproducibility analysis using five independently prepared aptasensors (the inset figure displays the *R*_ct_ values of the electrodes).

An increasing concentration of Stx1B was incubated with the developed aptasensor, and the EIS was carried out using 5 mM K_3_Fe(CN)_6_/K_4_Fe(CN)_6_ in 1× PBS, pH 7.4. The data were plotted as a Nyquist plot (Fig. 5a). An increase in the diameter of the semicircle in the Nyquist plot was observed with increasing concentration of Stx1B, indicating an increase in the R_ct_. This behavior can be attributed to the hindrance of the electron transfer to the electrode–electrolyte interface, as binding of the target molecules to the immobilized aptamer on the electrode surface blocks the approach of the redox molecule to the electrode surface. At the optimal packing density, the upright orientation of the aptamers provides better accessibility of the target molecule to the immobilized aptamer, leading to better sensitivity of the developed aptasensor. Furthermore, as the target concentration increases, the degree of surface coverage correspondingly rises, thereby enhancing the blocking effect and leading to a more pronounced suppression of redox probe access to the electrode. A linear increase in *R*_ct_ value was observed with increasing concentration of Stx1B over the range 10 pM to 450 pM, with the regression equation *y* = 19 817.44 + 107.02*x* and a correlation coefficient of 0.99 (Fig. 5b). The LOD of the sensor was determined to be 2.88 pM (*n* = 3), calculated using the equation LOD = 3 × SD/sensitivity, where SD represents the standard deviation of the measured R_ct_ value of the blank, and the sensitivity corresponds to the slope of the linear calibration curve.^32^

### 3.5 Performance evaluation of the developed aptasensor

The selectivity of the developed biosensor was evaluated to assess its ability to specifically detect the target molecule. The aptasensor was incubated with non-target molecules of nearly similar structure, as well as with random non-target molecules, to assess the sensor's discrimination capability. The sensor response obtained for these molecules was compared with that of the target molecule to evaluate the specificity of the aptamer–target interaction. The aptasensor was incubated with 250 pM of Stx1B, 400 pM of Stx2B, and 300 pM of Bovine Serum Albumin (BSA) individually for 20 minutes, and EIS was measured using a 5 mM K_3_Fe(CN)_6_/K_4_Fe(CN)_6_ solution in 1× PBS, pH 7.4.

The *R*_ct_ obtained after circuit fitting of the spectra were plotted. To negate the *R*_ct_ value of the blank, the Δ*R*_ct_ data were plotted, where Δ*R*_ct_ = (*R*_a_–*R*_0_)/*R*_0_, with *R*_0_ representing the *R*_ct_ value of the aptamer-immobilized aptasensor and *R*_a_ representing the value of *R*_ct_ with the Stx1B, Stx2B, and BSA incubations. From Fig. 5c, it is evident that a significant Δ*R*_ct_ value was obtained only for the target molecule, Stx1B, whereas there was no significant change observed for Stx2B and BSA, as compared to the blank aptasensor. The change in Δ*R*_ct_ value after incubating with Stx1B was due to the specific binding of the aptamer and Stx1B, confirming the high selectivity of the aptasensor.

The stability of a biosensor is a crucial parameter, as it directly determines the reliability, reproducibility, and practical usability of the sensor over time. A stable biosensor maintains its sensitivity, selectivity, and response signal over an extended period. To study the stability of the developed sT3 aptasensor, EIS measurements were done every 5th day over a 25 days period, and the changes in the EIS spectra and the *R*_ct_ value were recorded. During the study, the aptasensor was stored in 1× PBS solution with a pH of 7.4 at 4 °C. As shown in Fig. 5d, there was minimal deviation in the EIS spectra throughout the assessment, and the biosensor retained 97.6% of its initial response after 25 days. The high stability of the developed biosensor can be attributed to the strong covalent bonding between the gold and sulfur atoms of the electrode and the thiol-modified aptamer.

Furthermore, the reproducibility of the fabricated aptasensor was assessed by measuring the EIS response of five independently prepared aptasensors incubated with a 300 pM of Stx1B. The relative standard deviation (RSD) of the *R*_ct_ value was found to be 3.9%, indicating a reasonable reproducibility of the electrode fabrication process (Fig. 5e).

### 3.6 Real sample analysis with the developed aptasensor

To validate the applicability of the developed aptasensor in the real-world conditions, a recovery studies were performed using milk samples spiked with different concentrations of Stx1B. To minimize the potential influence of various matrices, present in the milk such as fats, proteins, the fat layer was removed by centrifugation. The milk sample spiked with Stx1B was incubated with the aptasensors for 20 minutes, and EIS measurements were performed using a 5 mM K_3_Fe(CN)_6_/K_4_Fe(CN)_6_ solution in 1× PBS, pH 7.4. Based on the measured *R*_ct_ value, the concentration of Stx1B in the sample was calculated using the calibration curve equation. The obtained recovery values range from 97.5% to 103.5%, indicating the developed aptasensor can efficiently detect Stx1B from milk samples (Table S3). The close agreement in the values obtained from the spiked milk sample and sample from the buffer, indicates effective mitigation of the matrix interference.

Table 3 summarizes the biosensors developed for detecting Stx1, highlighting their sensing performance and detection methodologies. The majority of the developed sensors are antibody-based, whereas only one aptamer-based sensor for the detection of Stx1 has been reported. Owing to the variation in the molecular nature of the target used by the reported sensors, a fair cross-comparison of the analytical performance of the sensors is challenging. Despite the differences in the molecular nature of the target used, the LOD of the reported sensors falls within a similar range.

**Table 3:** Biosensors reported for the detection of Stx1

Biosensor platform: biosensor type	LOD	Linear range	Sensitivity	References
Lateral flow assay: immunosensors	0.1 ng mL^−1^	2.5–20 ng mL^−1^	—	34
ELISA : immunosensors		10–50 pg mL^−1^	—	14
Voltammetry: immunosensors	2 ng mL^−1^		—	15
Voltammetry: aptasensor	0.044 ng mL^−1^^a^	0.05–100 ng mL^−1^^a^	5.0 µA ng^−1^ mL	35
Impedance-based: aptasensor (This work)	0.025 ng mL^−1^ (2.88 pM)	0.09–4.50 ng mL^−1^ (∼10 pM – 450 pM)	107.02 Ω pM^−1^	This work

a Normalized the unit for comparison purpose.

## 4. Conclusions

This work reports the development of a highly sensitive (picomolar range), label-free, and selective EIS-based aptasensor for Stx 1, and its performance was validated using real samples. The novel aptamer specific to Stx1 incorporated into the sensor was enriched from a randomized aptamer library, isolated, and systematically characterized through a combination of molecular techniques, computational analyses, and advanced instrumentation. In parallel, an interdigitated chain-type microelectrode was successfully fabricated as the sensing platform using advanced microfabrication techniques, including electron-beam deposition, photolithography, and wet-chemical etching. The resulting aptasensor demonstrated excellent operational stability and reusability, underscoring its potential for commercial application. This high functional stability can be attributed to the strong chemical conjugation between the aptamer and the sensor surface achieved through the SAM-based immobilization strategy. Moreover, the compact size of the interdigitated electrode (14 mm × 3.5 mm) expected to facilitate its integration with portable potentiostats, enabling its deployment prospects as a point-of-care (POC) device for rapid screening and detection of Shiga toxin-induced gastroenteritis, thereby supporting timely therapeutic intervention in affected regions.

## Author contributions

M. Mili: planning methodology, experimental investigation, data analysis, original draft preparation. S. R. Chinnadayyala: design and fabrication of electrodes, S. Cho: conceptualization of interdigitated microelectrodes, P. Goswami: project administration, supervision, reviewing and editing of manuscript.

## Conflicts of interest

The authors declare that they have no known competing financial interests or personal relationships that could have appeared to influence the work reported in this paper.

## Supplementary Material

RA-016-D5RA09084A-s001

## Data Availability

The data supporting this article have been included as part of the supplementary information (SI). Supplementary information: SDS-PAGE, western blot, CD, MALDI-TOF of Stx1B; agarose gel bands of aptamers after SELEX cycles; ITC profile of aptamer protein interaction; table of SELEX parameters; sequences of selected aptamers; recovery of the target protein from spiked milk samples. See DOI: https://doi.org/10.1039/d5ra09084a.
