# Supplementary material for: Development of an impedimetric aptasensor for Shiga-like toxin 1 using a DNA aptamer and an interdigitated microelectrode
Source: RSC Adv. 2026 Jan 21;16(5):4590–601. doi: 10.1039/d5ra09084a (PMC12821148; doi:10.1039/d5ra09084a)
Supplement: RA-016-D5RA09084A-s001 [file RA-016-D5RA09084A-s001.pdf]

Supplementary Information (SI) for RSC Advances.
This journal is © The Royal Society of Chemistry 2026

SUPPLEMENTARY INFORMATION

Development of an impedimetric aptasensor for Shiga-like toxin 1 using a DNA aptamer and interdigitated microelectrode

Malaya Mili[a], Somasekhar R. Chinnadayyala[b,1], Sungbo Cho[b,c], and Pranab Goswami*[a]

a. Department of Biosciences and Bioengineering, Indian Institute of Technology Guwahati, Guwahati 781039, Assam, India.
b. Department of Electronic Engineering, Department of Semiconductor Engineering, Gachon University, Seongnam-si, Gyeonggi-do, 13120, Republic of Korea.
c. Gachon Advanced Institute for Health Science & Technology, Gachon University, Incheon 21999, Republic of Korea.

Current address: Transdermal Diagnostics Ltd, Science Creates, Old Market, Midland road, Bristol, BS2 0NS, England, United Kingdom.

*Address for correspondence: pgoswami@iitg.ac.in (P. Goswami)

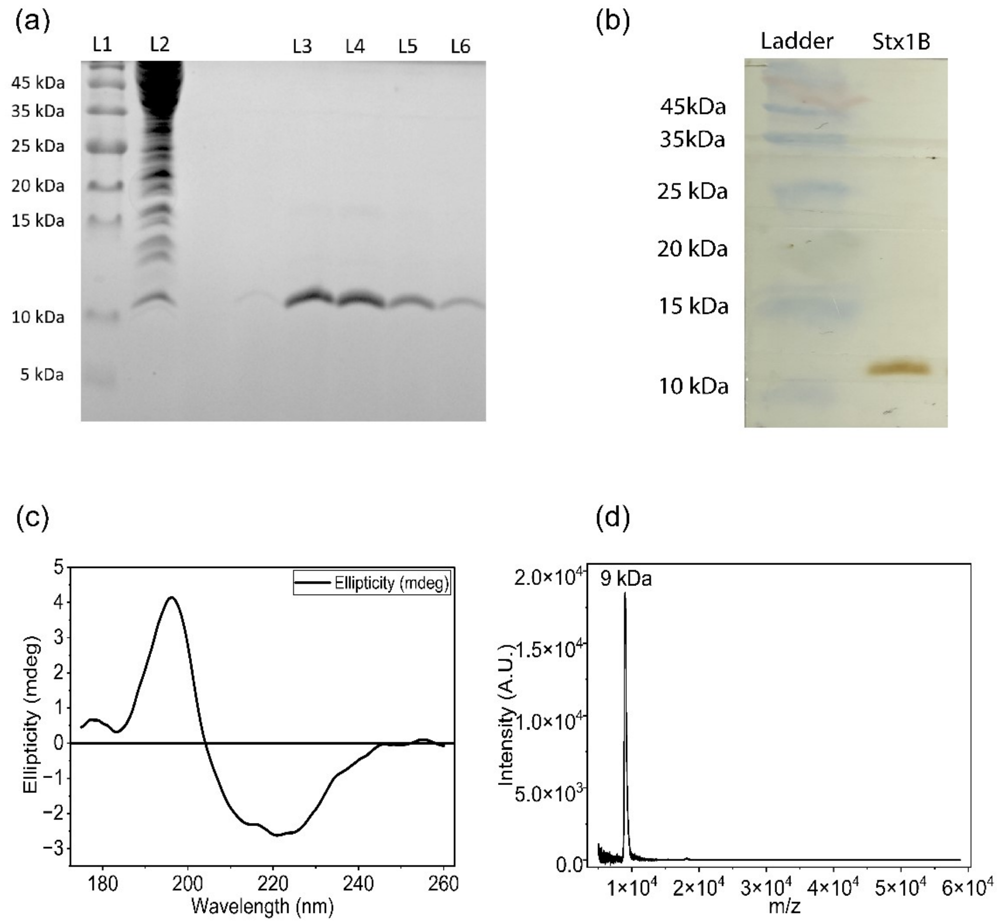

Figure S1: (a) Tricine SDS-PAGE analysis of purified Stx1B, L1- Ladder, L2- crude cell lysate, L3-L6- Purified Stx1B, (b) Western blot analysis of Stx1B using anti-Stx1B antibody, (c) CD

spectrum of Stx1B, and (d) MALDI-TOF analysis of Stx1B. All corresponding experiments for the characterization of Stx2B were also performed.

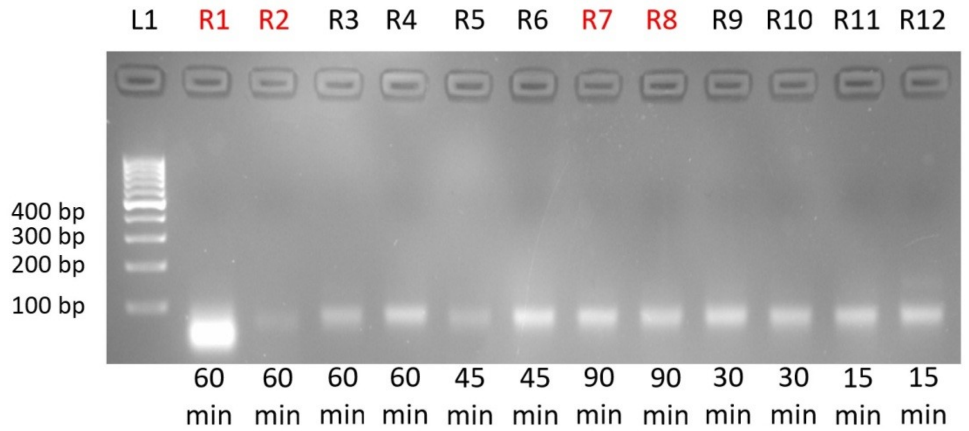

Figure S2: Amplified bands at the end of each SELEX round. The red rounds represent the negative SELEX. The incubation time for each round is indicated at the bottom of the gel.

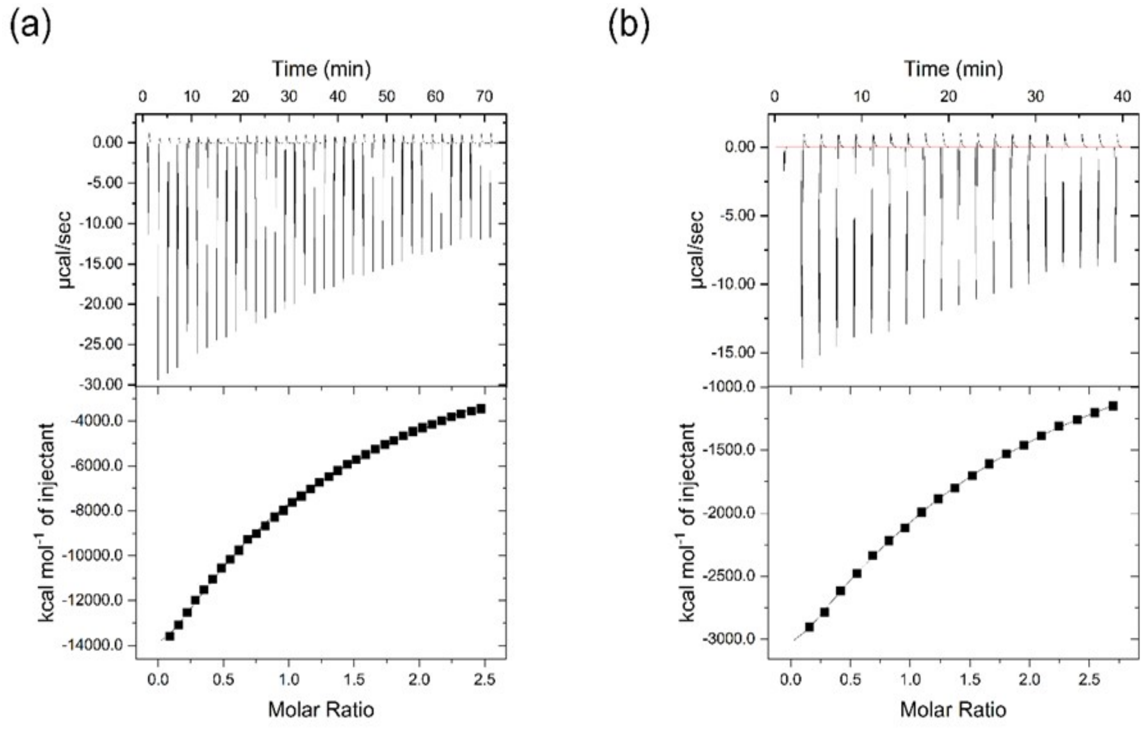

Figure S3- ITC profile of the interaction between (a) Stx1-sT3 and (b) Stx1-sT12.

Cycle number	Molecule incubated with the library	Positive/Negative SELEX cycle	Incubation time (Minutes)
R1	Ni-NTA beads	Negative	60
R2	Stx2B	Negative	60
R3	Stx1B	Positive	60
R4	Stx1B	Positive	60
R5	Stx1B	Positive	45
R6	Stx1B	Positive	45
R7	Ni-NTA beads	Negative	90
R8	Stx2B	Negative	90
R9	Stx1B	Positive	30
R10	Stx1B	Positive	30
R11	Stx1B	Positive	15
R12	Stx1B	Positive	15

Table S1: Selection parameters of SELEX process.

Sequence Name	Sequence (5'-3')
sT3	CACCTAATACGACTCACTATAGACTAAAGGGGCTTGATACCATAACTATG TCCTGGCTCGAACAAGCTTGC
sT12	CACCTAATACGACTCACTATAGTTGGCGCTGTATAGTCGAAGTAGTTACA TCCTGGCTCGAACAAGCTTGC

Table S2: Selected aptamer sequences.

Spiked	Detected	Recovery %	RSD%
10 pM	10.35 pM	103.5	0.97

200 pM	197.6	98.8	1.66
400 pM	390 pM	97.5	1.98

Table S3: Recovery of the target protein from spiked milk samples.
